# ATM mediated phosphorylation of CHD4 contributes to genome maintenance

**DOI:** 10.1186/2041-9414-2-1

**Published:** 2011-01-10

**Authors:** Aaron J Urquhart, Magtouf Gatei, Derek J Richard, Kum Kum Khanna

**Affiliations:** 1 Signal Transduction Laboratory, Queensland Institute of Medical Research, Brisbane, Queensland 4006, Australia

## Abstract

**Background:**

In order to maintain cellular viability and genetic integrity cells must respond quickly following the induction of cytotoxic double strand DNA breaks (DSB). This response requires a number of processes including stabilisation of the DSB, signalling of the break and repair. It is becoming increasingly apparent that one key step in this process is chromatin remodelling.

**Results:**

Here we describe the chromodomain helicase DNA-binding protein (CHD4) as a target of ATM kinase. We show that ionising radiation (IR)-induced phosphorylation of CHD4 affects its intranuclear organization resulting in increased chromatin binding/retention. We also show assembly of phosphorylated CHD4 foci at sites of DNA damage, which might be required to fulfil its function in the regulation of DNA repair. Consistent with this, cells overexpressing a phospho-mutant version of CHD4 that cannot be phosphorylated by ATM fail to show enhanced chromatin retention after DSBs and display high rates of spontaneous damage.

**Conclusion:**

These results provide insight into how CHD4 phosphorylation might be required to remodel chromatin around DNA breaks allowing efficient DNA repair to occur.

## Introduction

It is essential that human cells detect, signal and repair DNA damage in order to prevent chromosomal instability or malignant transformation. DNA double strand breaks can be induced by a number of agents including ionising radiation (IR), reactive chemical species and during endogenous DNA processing events such as DNA replication [1]. These breaks must be repaired in order to maintain cellular viability and genomic stability. Once a break has occurred, cells respond by recruiting DNA repair proteins to the DSB sites and initiate a complex DSB response pathway, which includes altered transcriptional and translational regulation, activation of DSB repair and cell cycle checkpoint arrest.

It is clear that chromatin restructuring in response to DNA damage is essential for initiation, propagation and termination of DNA repair and may even precede DNA end resection. This process opens the DNA allowing the recruitment of repair factors and the amplification of the checkpoint and downstream signals [2]. Consistent with this, the DNA damage response can be activated in the absence of exogenous DNA damage by the tethering of DNA damage response proteins to chromatin, demonstrating the importance of chromatin as a scaffold in the activation and amplification of the DNA damage response. In mammalian cells, the accumulation of any one of early response proteins MDC1, Mre11, Nbs1 or ATM is sufficient to achieve checkpoint activation [2]. This work extends the seminal discovery by Bakkenist and Kastan [3] that changes in chromatin structure can lead to ATM activation [3]. Whether ATM directly senses the disturbance in chromatin structure or requires an unidentified DSB sensor to transmit the signal is thus far unclear. Nonetheless, ATM kinase activity is a primary driving force for chromatin alterations emanating from DSB induction. Emerging evidence suggests that rapid IR-induced phosphorylation of H2AX and MDC1 by ATM serves as a recruiting signal for E3 ubiquitin ligase RNF8 [4-7]. RNF8 acts together with RNF168 to ubiquitinate histone H2AX and other chromatin proteins in the vicinity of the break [8-10]. The combined activity of these ligases is required for productive recruitment of repair proteins including 53BP1 and BRCA1. Notably, histone ubiquitination on chromatin surrounding the DSBs has recently been shown to mediate RNF8 and RNF168 dependent tra! nscripti on repression, which suggests the existence of cross talk between cellular processes mediated by these post-translational modifications [11].

**Figure 1:**
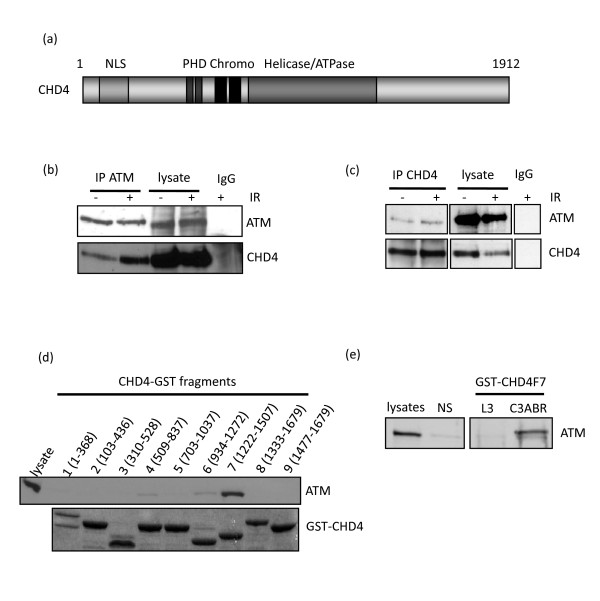
**CHD4 interacts with the DNA damage response kinase ATM**. (A) A schematic representation of the CHD4 protein, NLS; the putative nuclear localisation signal, PHD; plant homeodomain, Chromo; chromodomain, helicase and ATPase domain region. (B, C) ATM proficient C3ABR cells either mock irradiated or irradiated with 6Gy IR, 1hr after IR cell lysates were prepared and immunoprecipitated with for ATM, CHD4 or mock (IgG). The indicated proteins were subjected to western blot analysis. (D) Cell lysates prepared from C3ABR cells were subjected to pulldown assays using purified GST-CHD4 fusion protein fragments spanning the full length of CHD4. Upper Panel: ATM western blot of GST-CHD4 fusion fragment pulldown. Lower Panel: Coomassie stain of GST-CHD4 fusion fragments following SDS-PAGE. (E) ATM western blot following a GST-CHD4 fragment 7 pulldown or GST alone (NS) pulldown from L3 (ATM deficient) or C3ABR (ATM proficient) cell lysates.

CHD4 is a ≈210kDa protein that is highly conserved throughout the animal and plant kingdoms. The protein is composed of a PHD finger, two chromodomains and a C-terminal ATPase domain [12] and a putative C-terminal nuclear localisation signal (NLS) domain [13] (Figure 1a). The C-terminal ATPase/SNF-like helicase domain provides the energy required for histone displacement during nucleosome remodelling. Interestingly unlike other chromodomains that bind to histone marks, CHD4 has the unusual activity of binding directly to DNA [14]. CHD4 is a member of the class two family of CHD ATPases and is known to be a major subunit of the NuRD (nucleosomal remodelling and deacetylase) complex [15]. This complex has a number of enzymatic activities including chromatin remodelling, histone deacetylase and demethylase functions [13,15-19]. The loss of components of NURD complex leads to accumulation of ageing related chromatin defects [16]. Two recent publications have implicated CHD4 in the DNA damage response; specifically CHD4 depletion disrupts the chromatin response at the level of RNF168, preventing BRCA1 assembly [17,19]. CHD4 also functions as an important regulator of the G1/S transition by controlling p53 deacetylation as well as Cdc25A and p21^Cip1 ^stability [13,17]. This clearly implicates CHD4 as a novel chromatin-remodeling factor required for chromosomal stability.

Here we describe how CHD4 functions directly in the processes required for the efficient repair of DSBs. Our data demonstrates that CHD4 is phosphorylated rapidly at Ser-1349 by ATM following induction of DSBs and that this phosphoryated CHD4 is specifically located at sites of DSBs. We also demonstrate that the CHD4 Ser-1349 phosphorylation is required for the CHD4 chromatin retention and for the efficient repair of DSBs.

### Experimental procedures

#### Cell culture, plasmids, siRNA and antibodies

Human cell lines 293T, HeLa, CaCo2, U2OS, C3ABR, L3 and Seckle cells were maintained in RPMI 1640 medium supplemented with 10% foetal calf serum and incubated at 37°C with 5% CO_2_. Transfection of plasmids and siRNA was performed using Lipofectamine 2000 (Invitrogen) as per manufacturer's instructions. A series of GST-CHD4 constructs were generated, which together spanned the full length of CHD4. These constructs corresponding to F1 (1-1106), F2 (308-1307), F3 (930-1584), F4 (1528-2511), F5 (2109-3111), F6 (2802-3815), F7 (3666-4521), F8 (4000-5037) and F9 (4431-5037) were PCR amplified and then cloned into the GST-5x-3 vector (GE Healthcare) for expression. GST-CHD4 fusion proteins were purified as described previously [18]. Full length Flag-CHD4 plasmid was a kind gift from Yi Zhang. The Flag-CHD4 S1349A mutant was prepared by site-directed mutagenesis using the Quikchange mutagenesis kit (Stratagene) according to manufacturers instructions. RNA interference was performed using 5'-CAGUUACCAAGAAGACUUAdTdT-3' siRNA specific to CHD4 or control 5'UUCUCCGAACGUGUCACGUdTdT-3' siRNA. The siRNA sequences were synthesized as duplexes (Invitrogen).

Antibodies used in this study were supplied by: Cell Signaling (53BP1, Chk1 S317, p53 S15, ATM S1981), Oncogene (Brca2), Upstate (Acetyl-Lys-H4), Millipore (H2AX), Sigma (FLAG), Santa Cruz (p53, Chk1) GeneTex (ATM), anti-ATM AT9 for immunoprecipitation analysis was prepared in house [20]. The CHD4 antibody was a kind gift from Wei Dong. Rabbit polyclonal anti-phosphopeptide antisera against CHD4 (Serine 1349) was raised using the synthetic peptide NYNDG phospho-SQEDRDW-NH2 conjugated to keyhole limpet hemocyanin (KLH) at the Institute of Medical and Veterinary Sciences, Adelaide, Australia.

#### Immunoblotting and Immunoprecipitation

Cells were harvested in PBS and cell extracts were prepared by lysis in universal immunoprecipitation buffer (50 mM Tris-HCl, pH 7.4, 150 mM NaCl, 2 mM EDTA, 25 mM sodium fluoride, 25 mM β-glycerophsphate, 0.1 mM sodium orthovanadate, 0.1 mM phenylmethysulfonyl fluoride, 0.2% Triton-X 100, 0.3% Igepal CA-630, 0.5 mM dithiothreitol, protease inhibitor cocktail (Sigma)) and incubated on ice for 30 min and then sonicated with a Barnson Sonifier 450. Supernatants were collected following centrifugation at 14,000 × g for 15 mins. 25 μg of protein sample was then analysed by SDS- polyacrylamide gel electrophoresis and immunoblotting with appropriate antibodies. For co-immunoprecipitations protein samples were pre-cleared with protein A beads for 1 hr at 4°C. The supernatants were then incubated with the appropriate antibody for 2 hr. The immune complexes were collected with protein A and protein G beads. The complexes were washed twice with lysis buffer and then fractionated by SDS-polyacrylamide gel electrophoresis for immunoblot analysis. For protein pull-down assays, protein samples were pre-cleared with Glutathione Sepharose beads for 1hr at 4°C. The supernatants were then incubated with purified GST fusion protein fragments linked to Glutathione Sepharose beads for 2 hr. The complexes were washed twice with lysis buffer and then fractionated by SDS-polyacrylamide gel electrophoresis for immunoblot analysis.

**Chromatin Fractionation **was undertaken using a Sub-cellular Protein Fractionation Kit (Thermo Scientific Cat# 78840) as per manufacturer's instructions.

#### Kinase Assays

Cells were harvested in PBS and cell extracts were prepared by lysis in TGN buffer (50 mM Tris-HCl, pH 7.5, 50 mM β-glycerophsphate, 150 mM NaCl, 10% glycerol, 1% Tween 20, 1 mM NaF, 1 mM sodium orthovanadate, 1 mM phenylmethysulfonyl fluoride, 2 μg/ml pepstatin, 10 μg/ml aprotinin, 5 μg/ml leupeptin and 1 mM dithiothreitol) as described previously [21]. The immunoprecipitation was carried out as described above using an anti-ATM antibody. The immunoprecipitates were washed twice with TGN buffer, once with 100 mM Tris-HCl (pH 7.5) and 0.5 M LiCl, and twice with kinase buffer (10 mM Hepes, pH 7.5, 50 mM β-glycerophosphate, 50 mM NaCl, 10 mM MgCl_2_, 10 mM MnCl_2_, 5 mM ATP, and 1 mM dithiothreitol). Kinase reactions were prepared by resuspending washed beads in 30 μl of kinase buffer containing 10 μCi of [γ-^32^P] ATP and 1 μg of GST-CHD4 fusion protein. Immune complex reactions were incubated at 30°C for 30 min and analyzed by SDS-polyacrylamide gel electrophoresis followed by autoradiography.

**Immunofluorescence **was performed as described previously [18]. Briefly, cells grown on glass coverslips or on Ibidi 8 well μ-slides were washed with PBS, treated for 5 min with Triton buffer (0.5% Triton X-100 in 20 mM Hepes, pH 7.4, 50 mM NaCl, 3 mM MgCl2, and 300 mM sucrose) on ice to remove soluble proteins. Cells with the remaining chromatin-bound proteins were fixed with PBS-buffered 4% paraformaldehyde in 2% sucrose solution at room temperature for 10 min. Fixed cells were washed 3 times with TBS-T (25 mM Tris-HCl [pH 7.5], 150 mM NaCl, 0.1% Tween 20) and blocked in 3% BSA in TBS-T buffer for 30 min. Cells were then incubated with an appropriate primary antibody in blocking buffer at 4°C overnight. After washing three times in blocking buffer cells were incubated with the appropriate secondary antibody (Alexa 488 or Alexa 546, Invitrogen). Images were taken on a Deltavision PDV microscope or GE Healthcare In Cell Analyzer 2000.

#### In-nuclear-western

These assays were performed as described previously [22], using the GE Healthcare In Cell Analyzer 2000 and the data analysed using In Cell Investigator software. Assays were performed using GE Healthcare 96 well Matriplates. Immunofluorescence was performed as described above. The In Cell Analyser automatically counted 300 cells from each well prior to measuring the mean nuclear intensity for each signal (Alexa 488).

## Results

### CHD4 interacts with the ATM kinase

We originally identified CHD4 as an ATM interacting protein during co-precipitation (co-IP) experiments of GFP-tagged ATM and Flag-tagged CRM1. We consistently noticed an additional cross-reacting (Flag-antibody) band of ~200KDa in a GFP-ATM IP from irradiated cells. Bioinformatic analysis of proteins of similar size, in database, that show sequence similarity to Flag-epitope, identified CHD4 as a possible candidate. To confirm this initial observation we precipitated ATM from HeLa cellular extract before and after the induction of DSBs by IR (6 Gy) and blotted the immunoprecipitates with anti-CHD4 antibody. This confirmed that IR stimulated the interaction between ATM and CHD4 (Figure 1b). We also performed the reciprocal co-immunoprecipitation using CHD4 antibody followed by immunoblotting with anti-ATM and this also demonstrated that both proteins interacted (Figure 1c).

### CHD4 is phosphorylated by the ATM kinase

**Figure 2:**
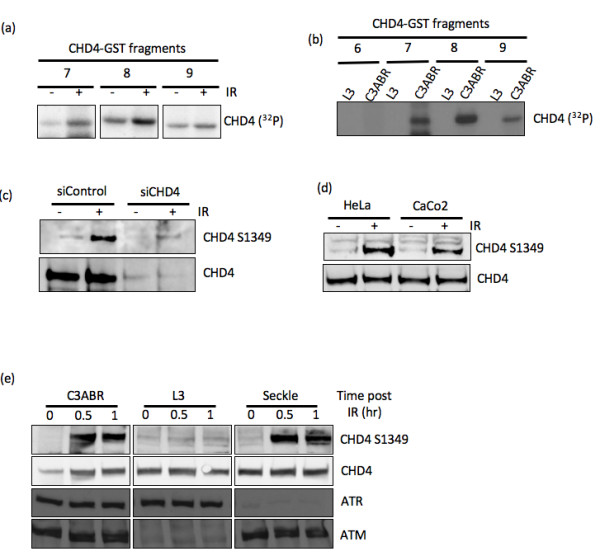
**CHD4 is phosphorylated in an ATM-dependent manner at Ser1349 following DNA damage**. (A) C3ABR cells were either mock irradiated or treated with 10Gy IR, 1hr after treatment cell lysates were prepared, immunoprecipitated for ATM and then an ATM kinase assay was performed against GST-CHD4 fusion protein fragments as indicated. (B) ATM proficient (C3ABR) and ATM deficient (L3) cells were irradiated with 10Gy IR, 1hr after IR cell lysates were prepared and immunoprecipitated for ATM and an ATM kinase assay was then performed against 1 μg of purified GST-CHD4 fusion protein fragments as indicated. (C) Mock (siControl) depleted or CHD4 depleted (siCHD4) U2OS cells were either untreated or treated with 6Gy IR, 1hr after IR cell lysates were prepared and subjected to western blot analysis for CHD4S1349 and CHD4 as indicated. (D) Hela cells and CaCO2 cells were either untreated or treated with 6Gy IR, 1hr after IR cell lysates were prepared and subjected to western blot analysis for CHD4S1349 and CHD4 as indicated. (E) ATM proficient (C3ABR) cells, ATM deficient (L3) cells and ATR deficient (Seckle) cells were either untreated or treated with 6Gy IR, cell lysates were then prepared at 30 min and 1hr after IR or from unirradiated cells. The lysates were then subjected to western blot analysis as indicated.

ATM is the apical kinase responsible for the initiation of cellular signaling following induction of DSBs [23]. It functions to phosphorylate a number of downstream substrates including p53, Chk2 and hSSB1 [24-26]. We initially mapped the ATM binding site on CHD4 using GST fusion fragments that span the full-length of CHD4. GST-CHD4 fragments bound to glutathione agarose were used to precipitate ATM from cellular extract [26]. We observed that CHD4 fragment 7 (representing amino acids 1222-1507) precipitated ATM from HeLa cellular lysates (Figure 1d). GST-pull down experiment with this fragment using cellular extracts from ATM-deficient (L3) and control (C3ABR) lymphoblastoid cells confirmed that the band detected in immunoblot was indeed ATM (Figure 1e). We next decided to determine if CHD4 is a substrate of the ATM kinase since it contains 8 consensus sites of phosphorylation by ATM (SQ/TQ). Using immunoprecipitated ATM kinase and the purified GST-CHD4 fragments as substrates we observed that phosphorylation of three C-terminal CHD4 fragments (Fragments 7-9) was induced after IR (Figure 2a). Furthermore, ATM immunoprecipitated from irradiated control (C3ABR) cells phosphorylated these fragments whereas immunoprecipitates obtained from L3 (not expressing ATM line, Figure 1e), failed to show any activity against each of the GST-CHD4 fusion proteins tested (Figure 2b). During this time, Ser-1349 in CHD4 was identified as an ATM phosphorylation site in a extensive phosphoproteomic screen of proteins precipitated by ATM phosphosite antibodies [27]. Of the three GST-CHD4 fragments phosphorylated by ATM in this study, Fragment 7 and 8 contain this phosphorylation site due to presence of overlapp! ing sequ ence between them. Next we raised phospho-specific antibody against Ser-1349 phosphorylated CHD4 peptide to monitor this phosphorylation event *in vivo *in cells. To confirm specificity of this antibody we immunoblotted control or CHD4 depleted U2OS extracts (using specific siRNAs) with or without prior exposure to IR (6 Gy) with antiphospho-specific antibody. CHD4 was found to be markedly phosphorylated in irradiated cell extracts from control siRNA transfected cell and this signal was diminished in CHD4-depleted cells (Figure 2c). This observed increase in IR-induced CHD4 phospho-Ser 1349 reactivity was also observed in HeLa and CaCO2 cell lines (Figure 2d). To confirm this was an ATM specific phosphorylation event we immunoblotted extract prepared from ATM-proficient (C3ABR), ATM-deficient (L3) and ATR- deficient (Seckle) cell lines with or without prior exposure to IR. While rapid IR-induced phospho-reactivity was observed in the C3ABR cells we were unable to observe induction in the L3 cells, which does not express ATM protein (Figure 2e), suggesting that this phosphorylation event was ATM specific. We also observed normal IR-induced CHD4 Ser-1349 phosphorylation in extracts from ATR-deficient Seckel cells. These observations confirm that ATM catalyzes rapid IR-induced phosphorylation of CHD4 on Ser-1349.

### ATM phosphorylated CHD4 (Ser1349) colocalises with γH2AX

**Figure 3:**
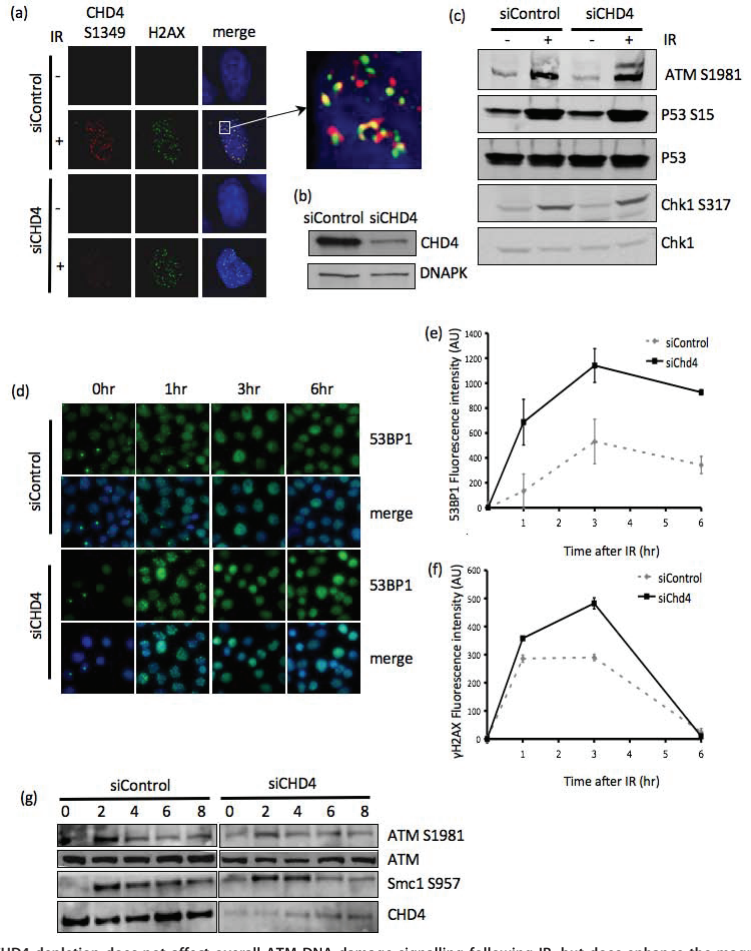
**CHD4 depletion does not affect overall ATM DNA damage signalling following IR, but does enhance the magnitude and duration of the DNA damage response**. (A) CHD4S1349 and γH2AX co-staining in unirradiated or 1 hr following 2Gy IR, in mock (siControl) and CHD4 depleted (siCHD4) HeLa cells. Dapi stained nuclei are shown in the merged image. Detergent pre-extraction was performed before immunostaining. The enlarged image shows CHD4S1349 and γH2AX co-staining. (B) HeLa cells were treated with mock (siControl) or CHD4 depleted (siCHD4), after 48 h cell lysates were prepared and subjected to western blot analysis for CHD4 and DNAPK as indicated. (C) HeLa cells were treated with mock (siControl) or CHD4 depleted (siCHD4). Cells were then treated with 10Gy IR or mock irradiated and cell lysates were prepared after 1 h. The lysates were subjected to western blot analysis for ATM S1981, P53 S15, P53, Chk1 S315 and Chk1 as indicated. (D) In-nuclear-western analysis of chromatin bound 53BP1 in mock (siControl) and CHD4 depleted (siCHD4) treated U2OS cells. Cells were treated with 4Gy IR or unirradiated (0 hr), at the indicated time points detergent pre-extraction was used to remove non-chromatin bound proteins prior to fixation and immunofluorescence analysis. Mean fluorescence signal was then calculated from at least 300 cells. (E, F) In-nuclearwestern analysis of chromatin bound γH2AX (E) and 53BP1 (F) in mock (siControl) and CHD4 depleted (siCHD4) U2OS cells treated as for (D). Graphs represent the mean of three experiments + and - the standard deviation. (G) U2OS cells were treated with mock (siControl) or CHD4 depleted (siCHD4). Cells were treated with 4Gy IR or unirradiated (0 hr), cell lysates were prepared at the times indicated and subjected to western blot analysis for ATM S1981, ATM, Smc1 S957, Kap1 S824 or CHD4 as indicated.

To explore the involvement of CHD4 in the DSB repair process we examined the nuclear distribution of CHD4 prior to and after treatment with IR. We were unable to observe any difference in the normal pan nuclear distribution of CHD4 following IR after staining with antibody that recognizes the total pool of CHD4 in cells (data not shown). Notably, using anti-phosphospecific CHD4 antibody (anti-Ser 1349), we found that phosphorylated CHD4 rapidly formed IR-induced (2 Gy) nuclear foci, which were found to co-localise with γH2AX, a known marker of sites of DSBs following IR (Figure 3a). This suggested that CHD4 is either specifically phosphorylated at these sites within the nucleus or that phosphorylated CHD4 re-distributes to these sites. This staining was specific as CHD4-depleted cells (Figure 3ab) showed a marked reduction in phosphorylated CHD4 nuclear foci. The depletion of CHD4, however, had no apparent effect on the γH2AX foci induction 30 minutes following IR (Figure 3ab). These results suggest that phosphorylated CHD4 may function in the process of DSB repair. Following the induction of DSBs, the MRN complex is recruited to the break sites [28]. There it stimulates the ATM kinase and amplifies downstream signaling [29]. Next, we determined the impact of CHD4-depletion on ATM activation and activity by assessing the autophosphorylation of ATM and phosphorylation of its downstream substrates. We were unable to observe any defect in rapid (1 hr after IR) activation of ATM signaling (Figure 3c). This would suggest that CHD4 is dispensable for the initial recruitment of MRN or ATM activation. Indeed, we were unable to observe any defect in Mre11 or Nbs1 recruitment to DSB repair foci in CHD4 depleted cells (siRNA) (data not shown).

The loss of chromatin remodeling has been shown to perturb the normal kinetics of the DSB repair process [22]. To examine if CHD4 regulates the normal processing of DSBs we determined the kinetics of loading of two well-established markers of the DSB repair, γH2AX and 53BP1 on to chromatin. Using in-nuclear-westerns [22], to measure chromatin bound 53BP1 and γH2AX, we observed that depletion of CHD4 (siRNA) resulted in a more pronounced recruitment of 53BP1 to chromatin (Figure 3de). The level of chromatin bound 53BP1 remained higher in the CHD4 depleted cells during the course of the experiment. γH2AX also showed a more rapid activation following IR; however, this was less marked than was observed for 53BP1 (Figure 3de, f). Interestingly, although ATM autophosphorylation (S1981) and SMC1 phosphorylation (S957) occurs in a timely manner in CHD4 depleted cells, these cells show a more rapid loss of signal (Figure 3g). These results are interesting as they demonstrate an abnormality in the repair kinetics of CHD4-deficient cells. It is possible that the loss of CHD4 function has a global effect on chromatin structure, effecting overall DNA repair protein accessibility of these damage sites and also exposing more DNA to damage. This could potentially result in higher rates of spontaneous damage as more DNA is exposed to the solute environment. However we failed to observe an increase in markers for spontaneous damage e.g. spontaneous γH2AX foci formation or spontaneous activation of ATM/ATR signaling events in CHD4-depleted cells. This may be due to technical limitation in achieving a complete knockout of CHD4 expression or that areas of DNA damage are rapidly repaired in these cells.

**Figure 4:**
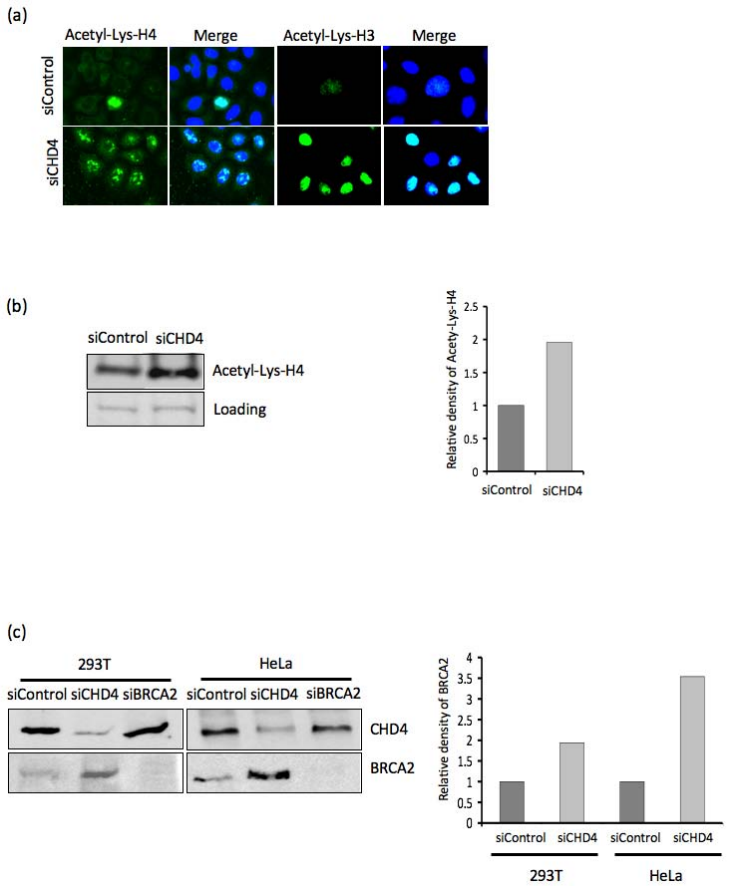
**CHD4 depletion leads to increased levels of euchromatin**. (A) U2OS cells were mock treated (siControl) or CHD4 depleted (siCHD4), 48 hr following treatment the cells were detergent extracted to remove non-chromatin bound proteins prior to fixation. Immunofluorescence analysis for Acetylated-lysine histone H4 (Acetyl-Lys-H4) and Acetylated-lysine histone H3 (Acetyl-Lys-H3) was then performed as indicated. The merge indicates co-staining with dapi stained nuclei. (B) U2OS cells were treated with mock (siControl) or CHD4 depleted (siCHD4), 48 hr following treatment cell lysates were prepared and subjected to western blot analysis for Acetyl-Lys-H4, densitometry was used to calculate the relative protein levels. Loading indicates a non-specific band common in each lysate. (C) 293T and HeLa cells were mock treated (siControl), CHD4 depleted (siCHD4) or BRCA2 depleted (siBRCA2), 48 hr following treatment cell lysates were prepared and subjected to western blot analysis for CHD4 and BRCA2 as indicated. Densitometry was used to calculate the relative levels of each protein.

It is likely that depletion of CHD4 results in a relaxation of chromatin structure, which would affect the ability of proteins to access the DNA structure and would also expose the DNA structure to the solute environment, causing DNA damaging events such as oxidation. This would explain the higher levels of chromatin bound 53BP1 and γH2AX observed following IR treatment in the CHD4-depleted cells. To determine if there was a global effect on chromatin structure in CHD4 depleted cells we used antibodies to euchromatin markers, Acetyl-lys-H3 and H4. These confirmed that there was a significant and general increase in the level of these markers in CHD4 depleted cells, indicating a higher degree of euchromatin (Figure 4ab). Microarray data of CHD4-deficient cells also indicated a global change in transcription levels, with over 10% of genes transcripts changing by >1.5 fold (1877 transcripts from an array of 18000 (data not shown). Consistent with this we observed cellular BRCA2 protein levels were elevated by over 3 fold in CHD4 depleted cells (Figure 4c). Importantly, increase in BRCA2 levels in CHD4-depleted Hela cells was not an artifact of cell cycle arrest, as this did not impact on normal cell cycle progression (data not shown).

### CHD4 phosphorylation by ATM is required for its recruitment to chromatin and the timely repair of DSBs following IR

**Figure 5:**
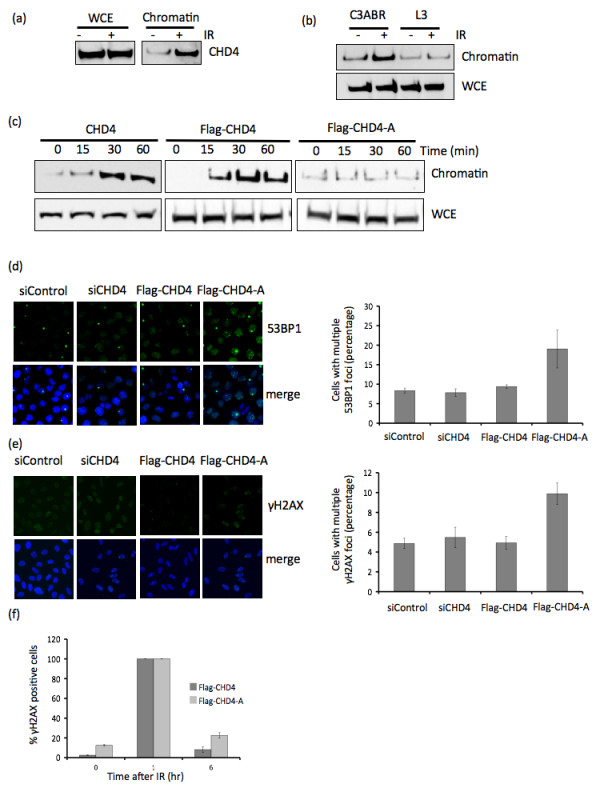
**CHD4 chromatin association is enhanced following DNA damage**. (A) HeLa cells we treated with 6Gy IR or untreated, 1 hr following IR treatment the cells chromatin fractionation analysis was performed. Whole cell extract (WCE) and chromatin fraction lysates were then analysed by western blot analysis with CHD4 as indicated. (B) ATM proficient (C3ABR) and ATM deficient (L3) cells we treated with 6Gy IR or untreated, 1 hr following IR treatment the cells chromatin fractionation analysis was performed. Whole cell extract (WCE) and chromatin fraction lysates were then analysed by western blot analysis with CHD4. (C) HeLa cells were either untransfected or transfected with plasmid DNA encoding Flag-CHD4 or Flag-CHD4 S1349A (CHD4-A). 24 hr after transfection the cells were treated with 6Gy IR. Cells were harvested at the times indicated and chromatin fractionation analysis was performed. WCE and chromatin fraction lysates were then subjected to western blot analysis with CHD4. (D, E) U2OS cells were either mock transfected or transfected with siCHD4 or with plasmid DNA encoding Flag-CHD4 or Flag-CHD4 S1349A (CHD4-A), 36 hr after transfection cells were detergent pre-extracted to remove non-chromatin bound proteins prior to fixation. Immunofluorescence analysis for 53BP1 (D) and (E) γH2AX was then performed and the percentage of cells (+ and - standard deviation) with multiple foci was calculated from a count of at least 100 cells across 3 independent experiments. (F) U2OS cells were transfected with plasmid DNA encoding Flag-CHD4 (CHD4) or Flag-CHD4 S1349A (CHD4-A), 24 hr after transfection cells were treated with 2Gy IR. Prior to IR and 1 hr and 6 hr after IR, cells were detergent pre-extracted to remove non-chromatin bound proteins prior then fixed for immunofluorescence analysis. The percentage of γH2AX positive cells (+ and - standard deviation) was then calculated from a count of at least 100 cells in 3 independent experiments.

Many DNA damage repair proteins become more strongly associated with chromatin following DNA damage. Since we have now shown that CHD4 is phosphorylated by ATM we decided to explore the chromatin association of CHD4 before and after DNA damage. Interestingly, we see a tighter association of CHD4 with chromatin following treatment with IR (Figure 5a). To determine if this association was ATM-dependent, we fractionated chromatin of ATM-proficient (C3ABR) and ATM deficient (L3) cells with or without prior exposure to radiation. While CHD4 loaded normally onto chromatin following IR treatment in ATM proficient cells, we did not observe increased IR-induced CHD4 chromatin loading in ATM-deficient cells (Figure 5b). We next decided to determine if damage-induced phosphorylation of CHD4 on Ser-1349 was required for its chromatin loading. Following IR, overexpressed Flag-CHD4 (wt) protein became more strongly chromatin associated, like endogenous CHD4, however the association of unphosphorylated Flag-CHD4-A (S1349A) did not change (Figure 5c). These data indicate that phosphorylation of CHD4, following DNA damage by IR, regulates it's association with chromatin. This phosphorylation is mediated by the ATM kinase and the S1349 site is essential for this process.

We have now established that CHD4 responds to DSB induction by IR. To determine if CHD4 is required for DNA damage repair we next analysed the appearance of spontaneous DNA damage in cells ectopically expressing Flag-CHD4 and the non-phosphorylatable Flag-CHD4-A. The ectopic expression of Flag-CHD4 had no effect on the number of γH2AX positive cells in the population, interestingly neither did the depletion of CHD4 (siRNA) which support the enhanced DNA repair phenotype observed earlier in this study. However, the ectopic expression of Flag-CHD4-A significantly increased the percentage of cells showing spontaneous (undamaged cells) γH2AX and 53BP1 foci (Figure 5de) suggesting that these cells had impaired ability to cope with endogenous DNA damage.

To further explore this we next treated HeLa cells with low dose IR (2Gy) to determine the repair kinetics of DSBs. To do this, we analysed the ability of HeLa cells expressing wt and phospho-mutant CHD4 to resolve γH2AX foci. Maximal γH2AX foci were observed in both wt-Flag CHD4 and Flag-CHD41349A mutant expressing cells at 1 h after irradiation. In wt-CHD4 transfected cells, γH2AX foci were present in ~ 7% of cells by 6 h after damage (Figure 5f). In contrast, Flag-CHD41349A mutant expressing cells were slower at resolving the γH2AX foci and retained γH2AX foci in ~20% of cells by 6 h which returned to predamage level 24 h after IR. These data indicate the phosphorylation of CHD4 at Ser 1349 is required for the efficient repair of double strand DNA breaks.

## Discussion

It is important to understand how cells respond to and attempt to repair double strand DNA breaks. Much is already known in regard to the recruitment of repair factors and the checkpoint activation pathways, however, it is becoming increasingly clear that chromatin remodeling must take place to allow efficient repair. CHD4 has previously been characterized as a component of the NuRD transcriptional repression complex [15,30] that exists as a part of a complex with ATM-related kinase ATR [31].

Here we identified CHD4, as an ATM interacting protein and subsequently showed that it is a direct target of ATM kinase. CHD4 is phosphorylated on Ser1349 *in vivo *in cells in an ATM dependent manner in response to IR. During the course of our study, another study reported CHD4 as a phosphorylation target of ATM kinase that supports our findings [13]. This study reported that association of CHD4 with DNA lesions occurs independently of ATM instead the authors found that it is dependent on poly (ADP-ribose) specific pathway. Another study reported significant enrichment in chromatin binding of CHD4 in response to IR, which could be explained in part by its retention directly at the sites of DNA damage [17]. Consistent with this report, we also observed ATM-dependent increase in chromatin retention of CHD4 after IR. Moreover, we found that Ser1349 phosphorylated CHD4 accumulated into IR-induced foci which colocalize with γH2AX, while staining with antibody that detects the total pool of CHD4 remained unchanged in response to IR suggesting that phosphorylated CHD4 might play a role in the regulation of DNA repair processes. Although CHD4 is not an essential component of the repair process, our data demonstrates that CHD4 functions to allow the timely repair of DNA damage, which is essential to maintain cellular viability. Consistent with this, CHD4-depleted cells show an elevated level of recruitment of DNA repair factors to chromatin following DNA damage. This however may be due to the general opening of the chromatin structure in cells depleted of CHD4, which would allow more rapid recruitment and would also result in elevated levels of DNA damage. Indeed, we demonstrate that markers of euchromatin increase significantly in cells depleted of CHD4 and there are large cellular changes in the transcriptome. This supports a general role of CHD4 and the NuRD complex in regulating global chromatin structure and expla! ins the global impact of CHD4 depletion.

To explore the possible role of CHD4 in DSB repair further we decided to study the role of the ATM mediated phosphorylation of CHD4 on Ser1349. Following IR treatment, CHD4 becomes more tightly chromatin associated in an ATM dependent manner, which might help localize CHD4 in close proximity to DNA lesions. It is likely that ATM modifies the cellular function of CHD4 specifically at sites of DSBs since DNA damage is known to cause retention of active ATM kinase in detergent-resistant nuclear fractions [32]. Cells ectopically expressing Flag-CHD4-A, which cannot be phosphorylated by ATM, fail to show enhanced chromatin retention after IR suggesting that ATM dependent phosphorylation might result in CHD4 becoming more tightly associated with the chromatin and this allows for the opening of the chromatin structure and repair. Consistent with this, cells ectopically expressing ATM-phosphosite mutant CHD4 (Flag-CHD4-A) displayed higher rates of spontaneous DNA damage and showed a defect in their ability to resolve IR induced γH2AX foci in a timely manner.

Collectively, our data demonstrates how phosphorylated CHD4 functions to facilitate the repair of DSBs. Understanding the cellular processes that control chromosomal stability are of vital importance. Our data provides evidence that CHD4, a component of NuRD complex is required to allow the timely repair of breaks and provide further support to the idea that there is an intimate link between ATM kinase and chromatin remodelling complexes involved in different aspects of chromatin dynamics in mammalian cells. Therefore, dissecting the interplay of ATM kinase and chromatin modifying activities has become a vital step towards understanding of DNA damage response. This data gives us interesting insight into the function chromatin remodelers may play in modulating the effect of chemotherapeutic drugs [33]. Furthermore, therapies that target the chromatin modifying enzymes are being investigated for the treatment of cancer [34,35].

## Competing interests

The authors declare that they have no competing interests.

## Authors' contributions

AU, MG and KKK contributed to all aspects of the project. AU and MG carried out the experiments, KKK conceived the study, and participated in its design, coordination and contributed towards writing of the manuscript. D.R. contributed towards writing of the manuscript. All authors read and approved the final manuscript.
